# Mice, men and the relatives: cross-species studies underpin innate immunity

**DOI:** 10.1098/rsob.120015

**Published:** 2012-04

**Authors:** Clare E. Bryant, Tom P. Monie

**Affiliations:** 1 Department of Veterinary Medicine, University of Cambridge, Cambridge, UK; 2 Department of Biochemistry, University of Cambridge, Cambridge, UK

**Keywords:** toll-like receptor, NLR, pattern recognition receptor, species immunity, immune evolution

## Abstract

The innate immune response is the first line of defence against infection. Germ-line-encoded receptors recognize conserved molecular motifs from both exogenous and endogenous sources. Receptor activation results in the initiation of a pro-inflammatory immune response that enables the resolution of infection. Understanding the inner workings of the innate immune system is a fundamental requirement in the search to understand the basis of health and disease. The development of new vaccinations, the treatment of pathogenic infection, the generation of therapies for chronic and auto-inflammatory disorders, and the ongoing battle against cancer, diabetes and atherosclerosis will all benefit from a greater understanding of innate immunity. The rate of knowledge acquisition in this area has been outstanding. It has been underpinned and driven by the use of model organisms. Information obtained from *Drospohila melanogaster*, knock-out and knock-in mice, and through the use of forward genetics has resulted in discoveries that have opened our eyes to the functionality and complexity of the innate immune system. With the current increase in genomic information, the range of innate immune receptors and pathways of other species available to study is rapidly increasing, and provides a rich resource to continue the development of innate immune research. Here, we address some of the highlights of cross-species study in the innate immune field and consider the benefits of widening the species-field further.

## 2. The beginnings of innate immunity

Without question, the immune system is an essential product of evolution. At its most basic level, the immune response can be split into two arms: the innate and the adaptive immune response. Innate immunity provides a non-specific and generalized response to infection resulting in the induction of a pro-inflammatory immune response. It is conserved across evolution and found, in varying forms, in all multi-cellular organisms. In contrast, the adaptive immune response appears to be the prerogative of vertebrates and results in the generation of specific protective immunity against pathogens. Adaptive immunity also results in the generation of immunological memory, thereby allowing a more rapid, and more robust, response to subsequent antigenic challenge. The two arms of the immune response are intrinsically linked with the innate response influencing the development of adaptive immunity [1].

The concept of innate immunity as we know it was first proposed by Charles Janeway in an address to the Cold Spring Harbor Symposium in 1989 [2]. Subsequent work by Jules Hoffman and co-workers in Strasbourg led to the discovery of the first innate immune signalling receptor (pattern recognition receptor, PRR), Toll, in the fruitfly *Drosophila melanogaster*. They demonstrated that the production of antimicrobial peptides in *Drospohila* depended on the induction of a signalling cascade via the activation of the receptor Toll [3]. The identification of the human homologue of the Toll protein (Toll-like Receptor 4, TLR4) by Janeway's group [4] paved the way for Bruce Beutler and co-workers to show that the TLR4 protein was the receptor for bacterial lipopolysaccharide (LPS) driving the inflammatory response to endotoxin in mice [5]. Specifically, they characterized a mutation, P712H, in the BB loop region of the TLR4 cytoplasmic signalling Toll Interlekin-1 receptor (TIR) domain of C3H/HeJ mice that rendered the mice resistant to the effects of LPS [5]. The seminal nature of these works and their subsequent impact on immunology was recognized by the awarding of the 2011 Nobel Prize in Physiology or Medicine to Jules Hoffman and Bruce Beutler, for identifying PRRs, jointly with the late Ralph Steinman for his discovery of dendritic cells [6]. The initial observations regarding the similarities between the immune systems of the fly and humans beautifully demonstrate the advantages of cross-species biology in developing our understanding of how the innate immune system works. The use of predominantly human, murine and *Drosophila* models has helped facilitate the growth of our understanding of the innate immune system at an unprecedented rate. In this paper, we address some of the successes of cross-species research in innate immunity, highlight some of the caveats, and provide examples of where other, less mainstream, species have already significantly benefited research in innate immunity.

## 3. Pattern recognition receptor: form and function

PRRs can be broadly classified into five different classes: TLRs, nucleotide-binding leucine-rich repeat-containing receptors (NLRs), retinoic acid-inducible gene-I (RIG-I)-like receptors (RLRs), C-type lectins (CTLs) and Absent-in-melanoma (AIM)-like receptors (ALRs). Together these receptor families provide an extensive repertoire of defence sentinels responsive to activating ligands from exogenous sources, such as pathogens and allergens, as well as endogenous danger signals. TLRs can be found in the plasma membrane, where they detect a wide variety of lipid or protein-based ligands. TLRs also reside in endosomal membranes, where they respond to nucleic acids, providing a key element of the antiviral response. CTLs are membrane-associated, albeit just on the plasma membrane. CTLs generally recognize carbohydrate-based ligands and are important sentinels for the detection of fungal infections. The NLRs, RLRs and ALRs are cytoplasmic receptors. The NLRs characterized to date respond to a variety of viral, bacterial and host-derived ligands. The RLRs and ALRs respond to viral RNA and bacterial or viral DNA, respectively. Hence, for many pathogens, a wide variety of different receptors and their signalling pathways will be simultaneously activated.

Receptor activation results in the induction of a pro-inflammatory immune response. This response is characterized and controlled by the production of pro-inflammatory cytokines such as tumour necrosis factor alpha (TNFα), interleukins (IL) -1 and -8, and interferon (IFN). The precise combination and quantities of cytokines produced by PRR activation will dictate the exact nature and severity of the immune response. For example, IL-8 is a strong chemoattractant for neutrophils and type I IFNs help to promote cellular defences against viral infection. IL-1 is a key mediator of inflammation, and inhibition of IL-1 signalling is of major interest for the treatment of many inflammatory and autoinflammatory conditions. Production of IL-1β during innate immunity is the prerogative of the inflammasome. The inflammasome is a multi-protein complex formed by PRRs such as NLRP3, NLRP1, NLRC4/NAIP (neural apoptosis inhibitory protein) and AIM2. In most inflammasomes, the adaptor protein ASC (apoptosis-associated speck-like protein containing a caspase activation and recruitment domain, CARD) is used to recruit procaspase 1. Procaspase 1 undergoes subsequent cleavage to release active caspase 1, which can then process pro-IL-1β and pro-IL-18 to facilitate secretion of the active cytokines from the cell. The detailed mechanisms of PRR activation, their signalling cascades and the resultant cellular effects have been widely reviewed [7–10].

Interestingly, there is marked variation in the range and number of PRRs possessed by different species throughout biology (table 1). Such variation underlies evolutionary pressures upon the developing immune system and may well be indicative of the types of threats commonly experienced by each species. Recent analysis of the evolution of the domain architecture in TLRs and NLRs identified a highly complex evolutionary history [11]. Zhang and co-workers concluded that in addition to clear evidence of species-specific receptor expansion, there has also been independent evolution of the protein folds used by these receptors [11]. Independent evolution across diverse species can lead one to assume that the major range of protein domains used in the innate immune system—LRR (leucine-rich repeat), CARD, PYD (pyrin domain), NACHT (domain present in NAIP, CIITA, HET-E and TP1), DD (death domain), TIR—provides the most suitable tertiary structures for the required cellular functions.

**Table 1.** The number of TLR and NLR family members varies between species. The number in parentheses for teleost TLRs reflects the paralogues identified in the Atlantic cod genome.

	TLRs	NLRs
*Drosophila*	9	0
human	10	22
murine	12	34
chicken	10	1
teleosts	17 (36)	NLR-A–5
		NLR-B–6
		NLR-C-hundreds
purple sea urchin	222	203
amphioxus	71	118

The most notable variation in PRR repertoire occurs between species with a divergent ancestry (table 1). *Drosophila* possesses nine orthologues of the TLR pathway. Of these only Toll itself has a fully confirmed role in innate immunity. Toll-9 has been implicated, via a genome-wide expression analysis, in the activation of innate immune signalling pathways, and may also potentially contribute to innate immunity in the fly. Activation of *Drosophila* Toll is through binding to the endogenous ligand Spätzle. Mammalian TLRs are primarily viewed as receptors for exogenous molecules such as LPS, although there is an increasing repertoire of endogenous ligands that have been reported [12]. Meanwhile, *Caenorhabditis elegans* has just a single TLR orthologue, Toll family protein 1 (TOL-1), which appears not to function in an innate immune capacity at all. Instead, this function falls to the Toll/IL-1R (TIR) protein [13]. TIR is homologous to the human SARM1 (sterile α-and armadillo-motif-containing protein-1), although human SARM1 is a negative regulator of TLR signalling [14] rather than an activator of innate immunity.

Of course, one must remember that some PRRs have also had developmental roles identified either instead of or in addition to their function in innate immunity. Indeed, the prototype PRR, *Drosophila* Toll, is important in dorsal–ventral patterning during embryogenesis. Similarly, *Drosophila* Toll2 and Toll5–9 are all believed to have roles in development [15]. For example, Toll8 is important for glycosylation in the embryonic nervous system, while Toll2 has an important function in cell adhesion and migration during larval development. The mammalian TLR family have not as yet had any developmental roles identified. In contrast, mammalian NLR proteins appear to segregate, at least partially, into proteins with immune functions and proteins with developmental functions. NLRP2 may well be involved in epigenetic regulation and has been associated with Beckwith–Widemann syndrome, a form of foetal overgrowth; NLRP5 deficiency in the mouse results in failure of the embryo to pass the two-cell stage; NLRP7 is associated with trophoblast development and may be linked with neonatal growth disorders and termination; whereas NLRP14 is expressed at high levels in human testes and is also found in murine ovaries (reviewed by Kufer & Sansonetti [16]).

## 4. Pattern recognition receptor conservation and diversification across species

In comparison with mammals, some species have undergone PRR expansion. Analysis of the sea urchin genome has identified 222 TLR-like and 203 NLR-like genes. The observation of NLR-like genes in the sea urchin *Strongylocentrotus* led to a reconsideration of the evolutionary beginnings of the NLR family [17–19]. Previously, the absence of NLR family members in either *Drosophila* or *C. elegans* [20] had led to the view that the evolutionary origin of the NLR family resided with the teleost fish. However, the presence of tripartite NLR-like proteins in the sea urchin and other anthozoan cnideria [21] demonstrates an earlier evolutionary origin. It now appears plausible that evolutionary precursors of the NLR family exist in basal metazoans. Recent reports of potential NACHT–LRR genes in mosquitoes and freshwater crustaceans suggest that the NLRs may indeed be present in ecdysozoans [21]. Of course, one must also remember that the NLR proteins bear a marked similarity to the plant disease resistance (R) proteins, in terms of both domain organization and function [22]. However, rather than suggesting that eukaryotic cells possessed a common ancestral gene prior to the splitting of animal and plant lineages, it appears that the similarity of the NLR and R proteins is the result of convergent evolution [20].

Teleost fish have, to date, had 17 distinct members of the TLR family identified [23]. Some of these are homologous to mammalian TLRs; however, seven of them appear distinct to the teleost. A number of members of the teleost family show evidence for the presence of paralogues. This is most apparent in the recently sequenced Atlantic cod [24]. Here, five paralogues are observed for each of TLR7, 8 and 9, and eight paralogues for TLR22. The basis of this expansion is unknown, but one could hypothesize that it reflects a need to respond either more robustly to a specific subset of pathogens or to a requirement for subtle differentiation between similar activatory ligands. The teleost fish have orthologues of both the NLRC and NLRP subfamilies. In addition, they also encode a unique teleost-specific subfamily containing hundreds of receptors, NLR-C [25]. The NACHT domain of the teleost NLR-C subfamily is most closely related to the mammalian NLRC3 NACHT. However, the N-terminus of the teleost proteins contains a range of effector domains, including Pyrin domains, which suggests a diverse downstream signalling network. Unlike other NLR proteins, the teleost NLR-C subfamily members contain a B30.2 protein interaction domain downstream of the LRR domain. The functional role of the B30.2 motif has yet to be elucidated, but is likely to diversify the range of proteins with which these NLR-C subfamily members can interact. This in turn may well enhance the functionality of the receptors by enabling the activation of a wider range of cellular signalling pathways. It may also help facilitate receptor cross-talk. Understanding the mechanisms of activation and signalling of the teleost PRRs will be enlightening, and the innate immune repertoire of the teleosts is rapidly being characterized [26].

Other vertebrates also display different PRRs to those found in mammals. This is exemplified by the chicken. The chicken possesses orthologues of TLR3, 4, 5 and 7; in addition, it also encodes for both TLR1 and TLR2. However, both chicken TLR1 and TLR2 are present as two paralogues that result from gene duplications from the TLR1/6/10 and TLR2 family of vertebrate receptors [27]. Two additional TLRs are also found in the chicken—TLR15 and TLR21. TLR21 seems shared between birds and fish, and in chickens is a TLR9 orthologue [28]. TLR15 has to date only been found in the avian population [29] and is one of the most polymorphic TLRs in the avian repertoire, along with avian TLR5 [30]. As yet the precise ligand for TLR15 has not been identified, but it is known to be bacterial in origin [31]. This is a good example of why cross-species biology continues to fascinate—what does avian TLR15 respond to? What does this tell us about the development and pressures of the avian innate immune system? And how, if at all, does it relate to the spread of zooanotic infections relevant to both animal and human health? Identification of the ligand for chTLR15 will require experimental clarification, but one could hypothesize that it may well show a degree of specificity for bacterial species commonly encountered by the chicken. In fact, the presence of TLR15 in the chicken, coupled with the loss of other TLRs found in mammals, may well be an example of evolution in action and reflect the range of pathogens associated with the chicken. A similar rationale could be provided to the expansions of PRRs in the sea urchin and NLRs in the teleost fish—the aquatic pathogen repertoire will be much different to that routinely encountered by mammals.

Even within mammals, we see clear differences in PRR possession. For example, humans have 10 TLRs and 22 NLRs, whereas mice have 12 and 34, respectively (table 1). Within the NLR family NLRP1, NLRP4, NLRP9 and NAIP all appear to have undergone expansion in rodents. In contrast, NLRP8 and NLRP13 have been lost from the rodent genome, while NLRP11 appears to be primate-specific [32]. AIM2 has only been reported in primates and rodent species [33]. A putative AIM2 gene has been identified in the equine genome, but not, at present, in other mammalian genomes. The limited distribution of AIM2 leads to the obvious question of why specific innate immune pathways develop in some closely related species but not in others. Dogs possess multiple mutations in their gene for NLRC4, a PRR that recognizes a number of bacteria, which result in the insertion of premature stop codons and a dysfunctional receptor [34]. The evolutionary pressures, or lack of, which have driven such mutations are unknown, but one can presume that pathogen exposure plays a role. Clearly, there is extensive variation in PRRs across species, and data from comparative work may well reveal important information about pathogen host specificity and evolutionary biology.

## 5. Structures and species

**Figure 1.**
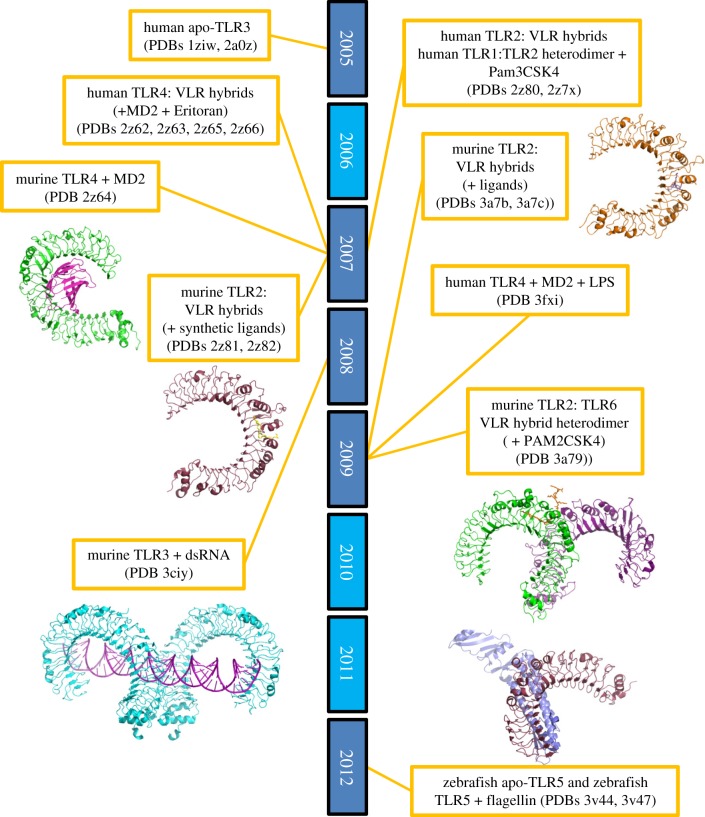
Timeline of TLR ectodomain structural characterization. The list to date of current structures of TLR ectodomains from humans, mice and zebrafish are shown in conjunction with their Protein Data Bank (PDB) identifies. Murine and zebrafish structures are presented in ribbon format and images were generated using the PyMOL molecular graphics system, v. 1.3, Schrödinger, LLC. Years highlighted in bright blue (2006, 2010, 2011) correspond to those in which no TLR ectodomain structures were published. PDB files are associated with the following references: PDB 1ziw [35]; PDB 2a0z [36]; PDBs 2z62, 2z63, 2z64, 2z65, 2z66 [37]; PDBs 2z80, 2z81, 2z82, 2z7x [38]; PDB 3ciy [39]; PDB 3fxi [40]; PDBs 3a79, 3a7b, 3a7c [41]; and PDBs 3v44, 3v47 [42]. VLR, variable lymphocyte receptor.

Determination of the molecular structure of pattern recognition receptors has proved to be difficult. Only in the last few years have we begun to understand the molecular detail involved in ligand recognition for the TLRs with the gradual solving of the apo- and ligand-bound forms of a selection of TLR ectodomains (figure 1). This began in 2005 when the apo-form of human TLR3 was solved independently by two separate research groups [35,36]. These structures provided the first experimental confirmation that the TLR LRR ectodomain did indeed form the type of solenoid-like structure that had been predicted. Producing sufficient quantities of purified protein for structural characterization has proved to be an arduous task for these proteins. It was another 2 years before any further TLR ectodomain structures were published. These were made feasible by the development of J.-O. Lee's work using variable lymphocyte receptor (VLR) capping techniques [43]. The VLR is an LRR-containing protein involved in the adaptive immune response of the sea lamprey. Following from the successful structural characterization of the VLR itself [44], the inspired approach of adding VLR capping structures onto the N- and C-termini, either individually or in parallel, of TLR ectodomains was initiated. This was feasible owing to the similar repeat size and consensus sequence between VLRs and TLRs [43,45]. The use of VLR capping technology has to date facilitated the high-resolution crystal structures of: human TLR4 in complex with MD2 and the antagonist Eritoran [37]; human and murine TLR2 in complex with various ligands [38,41]; a human TLR2:TLR1 heterodimer [38]; a murine TLR2:TLR6 heterodimer [41]; and, most recently, zebrafish TLR5 in complex with flagellin [42]. Interestingly, the structure of the active complex of TLR4:MD-2:LPS was solved without the need for VLR capping [40]. These structures provide a fantastic example of how merging protein sequences from different species can result in a hybrid protein conducive to downstream analysis, thereby significantly enhancing our biological understanding of TLR activation.

**Figure 2.**
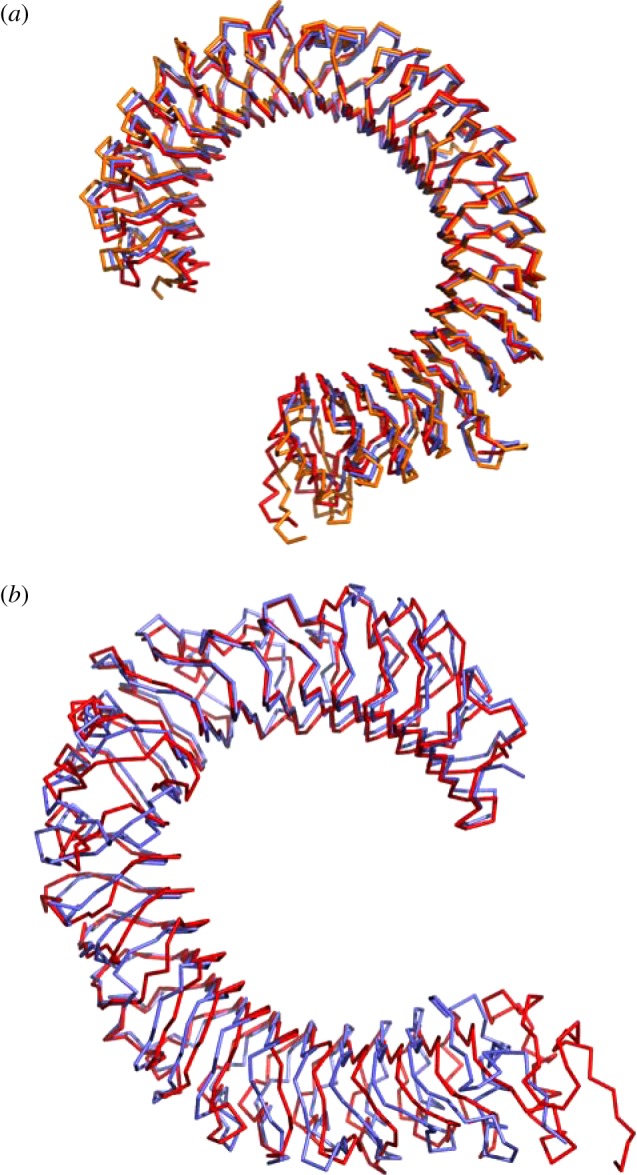
Murine and human TLR ectodomain structures are nearly identical. (*a*) Overlay of the ectodomains of murine TLR3 (PDB 3ciy, red) and both human TLR3 structures (PDB 2a0z, orange; PDB 1ziw, blue). (*b*) Overlay of the human (PDB 2z63, blue) and murine (PDB 2z64, red) ectodomains of TLR4. Structures are shown in a ribbon representation. Images were generated using the PyMOL molecular graphics system, v. 1.3, Schrödinger, LLC.

Solving the structures of murine, in addition to human PRRs is particularly enticing for two main reasons. First, a large proportion of PRR functional work is performed in murine cells. Second, the sequence identity between murine and human proteins is sufficient for the resultant structures to be highly similar. Consequently, they provide an excellent framework for understanding the molecular basis of receptor function in the absence of human information. This is highlighted by the structural similarities between the murine and human receptors of TLR3 and TLR4 (figure 2 and table 2). In fact, the TLR3 structures are so similar that the observed root mean square deviation of either of the solved human apo-TLR3 structures with the murine ligand-bound structure [39] is comparable to that between the two human structures (table 2).

**Table 2.** Root mean square deviations (r.m.s.d.) of murine and human TLR ectodomain structures. The PDB code of each molecule is given in parentheses.

molecule A	molecule B	r.m.s.d (Å)
human TLR3 (1ziw)	murine TLR3 (3ciy)	1.078
human TLR3 (2a0z)	murine TLR3 (3ciy)	1.521
human TLR3 (2a0z)	human TLR3 (1ziw)	1.083
human TLR4 (2z63)	murine TLR4 (2z64)	2.147

Other species are now being used for the determination of PRR molecular structures. As mentioned above, the crystal structure of zebrafish TLR5 in complex with the D1/D2/D3 fragment of *Salmonella* flagellin has just been solved [42]*.* Interestingly, during this work, Yoon and co-workers screened TLR5 from humans, mice, frogs, trout and zebrafish, but only the zebrafish construct resulted in the production of secreted, soluble protein in a baculovirus expression system. To generate sufficient quantities of protein for structural studies required subsequent addition of VLR capping structures.

Various domains of RIG-I have had their structures solved, both in the presence and absence of ligand [46]. However, the full-length receptor and the CARD domains had remained refractive to crystallization. This was recently resolved through utilization of RIG-I from *Anas platyrhynchos*, the mallard duck [47]. Duck RIG-I is 53 per cent identical to the human protein and shows good similarity in overall architecture of the protein in comparison with the human structures. This work has provided insight into the nature of RIG-I repression in the absence of ligand activation. CARD1 and CARD2 interact in a head-to-tail manner as a single unit. CARD2 subsequently binds to the helicase insertion domain, thereby inhibiting the binding of the RIG-I helicase domain to its double-stranded RNA ligand. It is postulated that this positioning of the CARD domains hinders the access of accessory proteins required to ubiquitinate RIG-I and facilitate signalling. Ligand binding by the C-terminal domain subsequently assists in the co-operative binding of ATP and double-stranded RNA to the helicase domain, the release of the CARDs, and the activation of RIG-I signalling [47]. Given the recent successes of zebrafish TLR5 and duck RIG-I, it is highly likely that a wider and more diverse range of species will be screened for permissiveness and suitability to structural studies in the next few years as more research groups try to resolve the molecular basis of innate immune function. This is particularly likely for receptors with little or no structural information, such as the NLRs.

## 6. Variation in ligand specificity between species

It is common knowledge that not all species respond to pathogen infections in the same way. This may relate to, for example, the site of infection, the symptoms that manifest, the severity of disease, the duration of infection, the host–pathogen relationship, the extent of the immune response, and the precise role of different ligands as agonists and antagonists.

The variation in the innate immune responses of different species to different ligands is beautifully highlighted by three different PRRs—TLR4, the NLRC4:NAIP inflammasome and NOD1. In the case of TLR4, which in conjunction with MD-2 responds to LPS [48], fascinating observations have been made regarding the different agonistic and antagonistic behaviour of ligands when recognized by human, mouse and horse forms of the receptor. The lipid A portion of *Escherichia coli* LPS is sufficient to induce TLR4:MD-2 activation. Lipid A molecules vary in the number and length of the acyl chains present, both as part of the biosynthetic pathways involved in lipid A production and also between the different types of lipid A produced by different bacterial species. This change in lipid A structure affects its immunostimulatory properties. For example, *E. coli* lipid A is primarily hexa-acylated and a strong receptor agonist across species. *Yersinia pestis*, the causative agent of plague, switches its Lipid A from an immunostimulatory hexa-acylated form to a non-stimulatory antagonistic tetra-acylated form at 37°C—a process that is believed to be an immune evasion strategy [49]. Different types of lipid A also induce different immune responses dependent on the species of receptor being activated. For example, the penta-acylated lipid A from *Rhodobacter sphaeroides* is an agonist in horses, and an antagonist in humans and mice [50]. Similarly, tetra-acylated lipid IVa from *E. coli* functions as an agonist in horses and mice, but as an antagonist to human TLR4:MD-2 activation [51,52].

Sequence variations in TLR4 and MD-2 between species can help explain the molecular basis of the species differences in TLR4:MD2 response to lipid A stimulation. For example, specific regions of horse MD-2 (residues 57–107) and horse TLR4 (LRR14 – 18) are required for the agonist activity of lipid IVa [52]. Furthermore, the agonistic activity of lipid IVa upon equine TLR4:MD-2 was lost when arginine 385 in equine TLR4 was mutated to a glycine, the equivalent human residue. When the crystal structure of human TLR4-MD2 and hexa-acylated lipid A was solved, it became apparent that the lengthy arginine sidechain could potentially stabilize the 1-phosphate group of lipid IVa, thereby enabling agonistic behaviour [40]. This story helps highlight how the combination of functional and structural studies from different species can provide essential insight into the mechanism of innate immune function. Zebrafish TLR4 appears not to respond to LPS, but instead negatively regulates NFκB signalling [53]. Should this be the case, it will be an interesting addition to the species-specific repertoire of TLR4 receptors that are helping to improve our understanding of the recognition of different types of bacterial LPS. Structural information derived from complexes of species-specific signalling receptors with their appropriate ligand will be important in explaining these data and will also aid the design of compounds to modulate TLR4 activity.

It is not just classical lipid-based TLR4 activation that shows species variation. TLR4 has also been implicated in the hypersensitivity reaction to the metal nickel [54]. Two histidine residues, H456 and H458, in the human TLR4 ectodomain are capable of inter-chelating with Ni^2+^ atoms, thereby activating the receptor in a lipid-independent manner and contributing to the induction of hypersensitivity. The murine TLR4 ectodomain lacks these two histidines and, as a consequence, mice do not experience nickel sensitization [54].

**Figure 3.**
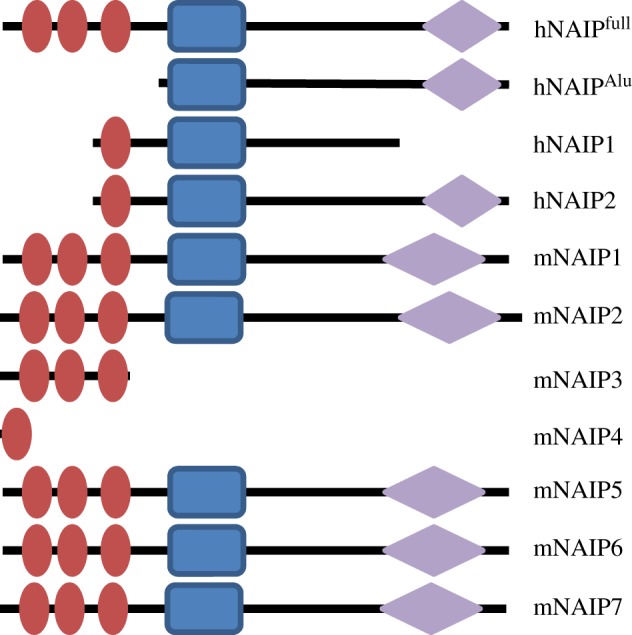
Predicted domain organization of NAIP proteins from different species. Red oval, BIR domain; blue rectangle, NACHT domain; lilac diamonds, LRR domain. Domain information derived from Ting *et al*. [57] and Romanish *et al*. [58].

The NLRC4:NAIP inflammasome provides an intriguing tale of species-specific behaviour in PRR biology. NLRC4 is a member of the NLR family that possesses an N-terminal CARD effector domain and forms an inflammasome complex that activates caspase 1 in response to stimulation with flagellin or type III secretion system proteins such as PrgJ from *Salmonella* [55,56]. Recently, the NAIP (also known as NLRB and Birc1; figure 3) proteins have been identified as a second group of NLR proteins involved in the formation of the NLRC4 inflammasome. It had been known for a while that murine NAIP5 was important for the control of, and immune response to, flagellin from *Legionella pneumophila* [59–61]*.* Subsequently, the groups of Vance and Shao have shown that different murine NAIP proteins provide the molecular basis for detection of specific ligands and signal through the formation of an inflammasome in conjunction with NLRC4 [62,63]. Specifically, murine NAIP2 is required for the detection of type III secretion components, while either murine NAIP5 or murine NAIP6 are capable of responding to flagellin. As yet the ligands, should there be any, detected by the remaining four mice NAIP proteins have not been identified. Murine NAIP3 and NAIP4 are reported to contain only baculoviral inhibition of apoptosis protein repeat (BIR) domains [57] and therefore may have a regulatory role, rather than one involved in detection.

It is currently difficult to directly relate the observations regarding the role of murine NAIPs to humans. The human genome contains one full-length gene for human NAIP and four partial deletions. While only a single full-length protein is believed to be expressed in human cells, various human NAIP isoforms have been detected as a result of the use of internal and upstream transcription start sites [58] (figure 3). The functionality of these isoforms remains unknown. What is clear is that humans do not possess clear orthologues of murine NAIP2, 5 and 6. Does human NAIP substitute for all of the functionality of murine NAIP2, 5 and 6? Certainly, we know that human NAIP has a role in the defence against *Legionella pneumophila* [64] and may therefore be able to mirror the role of murine NAIP5. Human NAIP has been shown to respond to the type III secretion apparatus of a variety of bacteria. However, it appears to be activated by the needle proteins rather than the rod proteins that stimulate murine NAIP2 [63]. The molecular basis of this specificity is as yet unknown. The functionality of the human NAIP:NLRC4 inflammasome and the role of human NAIP in the specificity of ligand detection remains a key unanswered question. Consequently, at least for now, one must be cautious in drawing parallels between the role of murine and human NAIP proteins. It will be intriguing to see how our understanding of the functionality of human NAIP develops. In addition to primates and rodents, NAIP orthologues have been identified in cows, horses and frogs. It may well be that the study of NAIP proteins from a wider range of species is required to fully understand the role of this PRR in innate immunity.

NOD1 is another member of the NLR family. Like NLRC4, it also possesses an N-terminal CARD effector domain. However, NOD1 responds to fragments of peptidoglycan from Gram-negative bacteria that contain a diaminopimelic acid moiety [65]. NOD1 does not form an inflammasome. Instead, it engages the CARD-containing kinase receptor interacting protein 2 (RIP2), resulting in the activation of NFκB responsive genes and the secretion of pro-inflammatory cytokines such as IL-8. Early studies on the receptor demonstrated that a while a tripeptide stem length is the optimal size for stimulating human NOD1, murine NOD1 is preferentially stimulated with a tetrapeptide stem length [66]. Although other lengths of peptide stem still activate the receptor, the proficiency of this response is reduced. In a follow-up study, mutagenesis identified the region most likely to be responsible for this species variation in optimal ligand length to a region around amino acids 816 and 844 [67]. In particular, mutation of the glutamic acid at position 816 in human NOD1, to either a serine or an aspartic acid (as found in the mouse), led to improved activation by the synthetic ligand FK156, consistent with the preference of murine NOD1. NOD1 from the pig, which, like the human sequence, has a glutamic acid at residue 816, shows a pattern of ligand responsiveness more closely matched to that of human NOD1 [68]. Comparative studies of NOD1 from a variety of species may help clarify the exact role of specific amino acids in the process of ligand recognition.

## 7. Inflammatory models of disease

Model systems are an essential part of immunological investigation. They provide a relatively accessible toolbox with which to probe our understanding of the genetic and molecular basis of immune signalling. Without question, they have contributed, and continue to contribute, extensively to our understanding of the human immune system. Both the fly and mouse were crucial in the early characterization of the immune system. Murine studies in particular have continued to expand our knowledge regarding the composition and functionality of the immune signalling pathways. Forward genetics and the generation of knock-out mice have enabled the precise role of individual components of the immune system to be determined. For example: the recognition of TLR9 as the cellular receptor for bacterial DNA [69]; the critical role of MyD88 in interleukin signalling [70]; the *in vivo* role of TLR2 [71]; the identification of the TLR adaptor protein TRIF [72]; the importance of NAIP5 for resistance to *Legionella pneumophila* infection [60]; and the importance of TLR9 in the sensing of unmethylated DNA as a protection against viral infection [73].

Studies in the mouse can be extremely enlightening, but they are not humans, and there are many differences in the immune systems, and therefore many limitations to mouse studies. The PRR make up of mice differs from that of humans (table 1). This will influence their response to pathogens and ligand stimulation, as well as the cross-talk and redundancy between PRR signalling pathways. The possession of additional paralogues (e.g. NAIPs, NLRP1, NLRP4 and NLRP9) complicates the generation of knock-out models, but also the interpretation of results and the direct relevance of inferred cross-species function. Even cases where receptor expression and copy number are identical may prove to be more complex. For example, both mice and humans express TLR8. In humans, TLR8 responds to small synthetic ligands such as imiquimod and R848, as well as single-stranded RNA. Murine TLR8, however, was believed to be non-functional owing to its failure to respond to similar ligands. This changed when Gorden and co-workers demonstrated that murine TLR8 transfected into HEK cells was indeed functional, but appeared to require a combination of polyT oligonucleotides and a small molecule human TLR8 agonist such as 3M-002 or 3M-003 (which also stimulates TLR7) [74]. More recently, the functionality of murine TLR8 has been further characterized by the observation that it mediates detection of poly (A)/T-rich DNA by plasmacytoid dendritic cells during poxviral infection [75]. A computational comparative analysis of TLR8 models from human, bovine, porcine, rat and mouse has now suggested a molecular basis for the inability of rodent TLR8 receptors to signal in response to small molecule ligands [76]. This analysis suggested not that rodent TLR8 was unable to bind to ligands such as R848, but that instead the affinity of interaction would be insufficient for activation of the receptor. Poly (A)/T-rich DNA consequently provides a specific role in enabling activation of rodent TLR8. The basis of this behaviour is hypothesized to relate to the predicted electrostatic charge differences in the region between LRRs 14–17 between species. This region includes a region of undefined structure. This undefined region shows poor sequence similarity between non-rodent TLR8s and may account for some of the differences seen in ligand specificity between these species [76]. It remains to be seen whether these observations will require the re-evaluation of models in which murine TLR8 was assumed to be non-functional.

Interesting observations have recently been made in relation to disorders in the cryopyrin-associated periodic syndromes (CAPS) spectrum of diseases. CAPS are characterized by three major diseases: familial cold auto-inflammatory syndrome (FCAS), Muckle-Wells syndrome (MWS) and neonatal–onset multi-system inflammatory disease (NOMID). Each of these autoinflammatory conditions results from the presence of polymorphisms in the NLR-family member NLRP3. Numerous polymorphisms have been linked with these diseases. Some of these mutations in humans are linked to just a single disease phenotype (for example, L353P and FCAS, A352V and MWS). Others associate with multiple phenotypes, such as R260W, which is linked with both FCAS and MWS. These polymorphisms result in constitutive activation of the NLRP3 inflammasome, caspase-1 processing, and secretion of IL-1β and IL-18. In humans, the diseases become more severe in the order FCAS, then MWS and finally NOMID. Recently, the first murine models of CAPS have been generated for mice with the mutations equivalent to human R260W, A352V and L353P [77,78]. These mice have provided the first opportunity to study the mechanistic basis and clinical changes associated with these diseases away from the use of patient-derived samples. The mice strains exhibited a more severe disease phenotype than humans overall, but without manifesting all human symptoms. These included inhibited growth, cutaneous lesions, abscesses and dermal thickening. Disease in the mice was shown to drive a T helper 17-type response and to be partially dependent on IL-1β, and hence inflammasome activation. Interestingly, the IL-1β dependence was less extensive than would have been predicted given the success of IL-1 inhibitors in treating CAPS [77,78]. This may reflect underlying differences in the murine and human manifestations of the diseases. It certainly causes one to consider how accurately observations in the mouse can be mapped onto the human patient. A related and intriguing observation is that in the work of Brydges and co-workers [78], the severity of murine disease showed a reversed order in comparison with humans CAPS. In humans, MWS is more severe than FCAS. However, mice harbouring the FCAS-specific knock-in showed a disease phenotype more severe than those harbouring the MWS-specific mutation. MWS-specific mice generally displayed symptoms at, or within, a day of birth, while the FCAS mutation usually led to perinatal or intrauterine lethality. The increased severity of these diseases in the mouse, and particularly the reversal in relative severity, raise some interesting questions, such as: how conserved is the structural environment of the mutation? Are there different co-factors recruited to human and murine inflamamsomes? Does the mechanism of inflammasome regulation, and auto-activation, differ between species? Are there other significant contributory factors to the pathology of CAPS? What underlies the molecular basis of this species-specific difference in disease phenotype remains unknown, and realistically requires structural information regarding the specific molecular environments of these mutations in both the mouse and human. In addition, an increased understanding about protein and/or co-factor interactions around the polymorphisms may assist in the unravelling of this observation.

The reversed phenotype of the CAPS mice, and the clear difference in the extent of response to IL-1 antagonism, highlights that while such models can clearly provide an unparalleled resource, the information obtained must be carefully analysed in light of species-specific differences. This is of particular relevance in the development of therapeutics against inflammatory conditions where modulation of the immune system is critical.

## 8. Final comments

The rapid development of our understanding of the functionality of the innate immune system could not have been predicted. It continues to advance at an awe-inspiring rate and the complexities of the system are only now beginning to become apparent. The regulation, the redundancy, the cross-talk, the increasing receptor repertoire, the contribution to adaptive immunity—these are just some of the major areas of ongoing development. Species-specific research has underpinned many of the scientific advances in this field over the last 20 or so years. Without question, we would not have such a clear understanding of the basis of innate immune functionality without the work performed on both the fly and the murine innate immune systems. Of course, humans are neither flies nor mice, and consequently, while in many cases these studies provide accurate and informative interpretation, we must always keep in mind that our immune systems do not function in precisely the same way as these other organisms.

With the increase in genome-sequencing projects, the information available on innate immune components in other species is rapidly expanding. This is a rich vein of information and we need to ensure that it does not simply become consigned to an electronic repository and forgotten. As yet, there is no concerted effort to curate the immune genes in these non-mainstream species, and we are reliant upon the valuable work of small groups of researchers specifically interested in subsets of genes for our increased understanding of their function. In addition, it will require time to enable the development of species-specific tools and reagents to facilitate comprehensive characterization of immune genes in non-model organisms. However, to date, we have only explored a small fraction of the available species pool. Who knows what marvels will be uncovered, and from what sources, in the near future?
